# Adverse childhood experiences are associated with the risk of lung cancer: a prospective cohort study

**DOI:** 10.1186/1471-2458-10-20

**Published:** 2010-01-19

**Authors:** David W Brown, Robert F Anda, Vincent J Felitti, Valerie J Edwards, Ann Marie Malarcher, Janet B Croft, Wayne H Giles

**Affiliations:** 1 Centers for Disease Control and Prevention, Atlanta, Georgia, USA; 2 Netherlands Institute for Health Sciences, Erasmus University Medical Center, Rotterdam, the Netherlands; 3 Southern California Permanente Group (Kaiser Permanente), San Diego, California, USA

## Abstract

**Background:**

Strong relationships between exposure to childhood traumatic stressors and smoking behaviours inspire the question whether these adverse childhood experiences (ACEs) are associated with an increased risk of lung cancer during adulthood.

**Methods:**

Baseline survey data on health behaviours, health status and exposure to adverse childhood experiences (ACEs) were collected from 17,337 adults during 1995-1997. ACEs included abuse (emotional, physical, sexual), witnessing domestic violence, parental separation or divorce, or growing up in a household where members with mentally ill, substance abusers, or sent to prison. We used the ACE score (an integer count of the 8 categories of ACEs) as a measure of cumulative exposure to traumatic stress during childhood. Two methods of case ascertainment were used to identify incident lung cancer through 2005 follow-up: 1) hospital discharge records and 2) mortality records obtained from the National Death Index.

**Results:**

The ACE score showed a graded relationship to smoking behaviors. We identified 64 cases of lung cancer through hospital discharge records (age-standardized risk = 201 × 100,000^-1 ^population) and 111 cases of lung cancer through mortality records (age-standardized mortality rate = 31.1 × 100,000^-1 ^person-years). The ACE score also showed a graded relationship to the incidence of lung cancer for cases identified through hospital discharge (*P *= 0.0004), mortality (*P *= 0.025), and both methods combined (*P *= 0.001). Compared to persons without ACEs, the risk of lung cancer for those with ≥ 6 ACEs was increased approximately 3-fold (hospital records: RR = 3.18, 95%CI = 0.71-14.15; mortality records: RR = 3.55, 95%CI = 1.25-10.09; hospital or mortality records: RR = 2.70, 95%CI = 0.94-7.72). After *a priori *consideration of a causal pathway (i.e., ACEs → smoking → lung cancer), risk ratios were attenuated toward the null, although not completely. For lung cancer identified through hospital or mortality records, persons with ≥ 6 ACEs were roughly 13 years younger on average at presentation than those without ACEs.

**Conclusions:**

Adverse childhood experiences may be associated with an increased risk of lung cancer, particularly premature death from lung cancer. The increase in risk may only be partly explained by smoking suggesting other possible mechanisms by which ACEs may contribute to the occurrence of lung cancer.

## Background

The Adverse Childhood Experiences (ACE) Study is a collaborative effort between Kaiser Permanente (San Diego, CA) and the Centers for Disease Control and Prevention (Atlanta, GA) designed to examine the long-term relationship between adverse childhood experiences (ACEs) and a variety of health behaviours and health outcomes in adulthood. An underlying thesis of the ACE Study is that stressful or traumatic childhood experiences have negative neurodevelopmental impacts that persist over the lifespan and that increase the risk of a variety of health and social problems [1].

Strong, graded relationships have been reported between traumatic stress during childhood and smoking behaviour [2,3]. Anda and colleagues [2] hypothesized that observed associations between adverse childhood experiences (ACEs) and early smoking initiation (by age 14 years) as well as other smoking behaviours in adulthood may be partly explained by the adoption of smoking as a means of self-medicating to deal with affective disorders through the psychoactive actions of nicotine. The epidemiological findings parallel advances in the neurobiological understanding of tobacco dependence [4-6] as well as that for the consequences of exposure to childhood traumatic stressors [7], including cancer [8], providing biologic plausibility to observed associations between child maltreatment and adverse health outcomes later in life [9-12].

For example, evidence from animal models, clinical studies, and neuroimaging studies suggest that child maltreatment affects brain regions (e.g., hippocampus, amygdala, and prefrontal cortex) and circuits such as the hypothalamic-pituitary-adrenal (HPA) axis and norepinephrine systems which mediate stress response [11]. Early stressors may have lasting effects on the HPA axis perhaps by increasing glucocorticoid response to subsequent stress [11]; that is to say, early life stressors may lead to sensitization of central nervous system corticotrophin releasing factor (CRF) activity [13]. Furthermore, disruptions in HPA signaling may sustain inflammatory processes (processes shown to have a role in the development of some cancers [14]) through altered release of glucocorticoid hormones and disturbances in the balance between pro- and anti-inflammatory mechanisms thereby affecting immune activation and inflammation [15-17].

The negative health consequences of smoking and second hand smoke exposure are well documented [18,19]. Smoking is responsible for at least 30% of all cancer deaths, for nearly 80% of deaths from chronic obstructive pulmonary disease as well as early cardiovascular disease and deaths [18]. An estimated 443,000 Americans die from diseases directly related to cigarette smoking each year [20], and smoking is estimated to be responsible for more than 5 million deaths per year worldwide [21]. Lung cancer, one of many smoking-related diseases for which evidence is sufficient to infer a causal relationship, is a leading cause of cancer death among both men and women in the United States. In 2005, 90,141 men and 69,079 women died of lung cancer in the United States [22].

On the basis of this evidence, we conducted a prospective cohort study using data from the ACE Study and ACE Mortality Study to examine the cumulative effect(s) of ACEs on the risk of lung cancer with particular attention given to an important causal intermediate, smoking behaviour. The *a priori *hypothesis was that ACEs are associated with an increased risk of lung cancer and that this relationship would operate through the ACE-smoking relationship.

## Methods

### Baseline cohort

The ACE Study methods have been described in detail elsewhere [1,2,7]. The ACE Study has been approved by the institutional review boards of the respective institutions. Briefly, the ACE Study is based at Kaiser Permanente's San Diego Health Appraisal Clinic, a primary care clinic where each year more than 50,000 adult members of the Kaiser Permanente Health Maintenance Organization receive an annual, standardized, biopsychosocial medical examination [2]. Each member who visits the Health Appraisal Clinic completes a standardized medical questionnaire [1]. The medical history is completed by a health care provider who also performs a general physical examination and reviews laboratory test results with the patient [1]. Appointments for most members are obtained by self-referral with 20% referred by their health care provider [1]. A review of Kaiser Permanente members aged 25 years or older in San Diego and continuously enrolled between 1992 and 1995 revealed that 81% of those members had been evaluated at the Health Appraisal Clinic [1].

All Kaiser members who completed medical examinations at the Health Appraisal Clinic between August and November of 1995, between January and March of 1996 (Wave I: 13,494 persons), and between April and October of 1997 (Wave II: 13,330 persons) were eligible to participate in the ACE Study [23]. Within two weeks after a member's visit to the Health Appraisal Clinic, a Study questionnaire was mailed asking questions about health behaviours and adverse childhood experiences. A total of 17,421 (68%) persons responded; 84 persons had incomplete information on race and educational attainment leaving 17,337 persons available in the baseline cohort [23]. Select characteristics of the baseline sample are shown in Table 1.

**Table 1:** Select characteristics of 17,337 ACE Study participants at baseline

Characteristic	N (%)
Age (years)	
18-34	1721 (9.9)
35-49	4494 (25.9)
50-64	5534 (31.9)
65-74	3715 (21.4)
≥ 75	1873 (10.8)
Women	9367 (54.0)
Nonwhite	4373 (25.2)
Education	
< high school	1251 (7.2)
High school graduate	3044 (17.6)
Some college	6220 (35.9)
College graduate	6822 (39.3)
Unmarried	5331 (30.7)
Financial problems	2040 (11.8)
Smoking status	
Current	1490 (8.6)
Former	7040 (40.6)
Never	8807 (50.8)
History of chronic obstructive pulmonary disease	781 (4.5)
History of asthma	1780 (10.3)
History of tuberculosis	1921 (11.1)

#### Definitions of Adverse Childhood Experiences (ACEs)

Adverse childhood experiences include childhood emotional, physical, or sexual abuse and household dysfunction during childhood. The categories are verbal abuse, physical abuse, contact sexual abuse, a battered mother, household substance abuse, household mental illness, incarcerated household members, and parental separation or divorce (Table 2). The experiences chosen for study were based upon prior research that has shown to them to have significant negative health or social implications, and for which substantial efforts are being made in the public and private sector to reduce their frequency of occurrence.

**Table 2:** Definition and age-standardized prevalence of adverse childhood experiences (ACEs) at baseline by smoking: Kaiser Permanente, San Diego, California, 1995-1997

	Ever Smoked, % (n = 8551)	Never Smoked, % (n = 8786)
**Childhood Abuse**		
*Emotional*	16.0	10.4
(Did a parent or other adult in the household ...)		
1) Often or very often swear at you, insult you, or put you down?		
2) Sometimes, often, or very often act in a way that made you that you might be physically hurt?		
*Physical*	36.9	26.1
(Did a parent or other adult in the household ...)		
1) Often or very often push, grab, slap, or throw something at you?		
2) Often or very often hit you so hard that you had marks or were injured?		
*Sexual*	27.3	19.2
(Did an adult or person at least 5 years older ever ...)		
1) Touch or fondle you in a sexual way?		
2) Have you touch their body in a sexual way?		
3) Attempt oral, anal, or vaginal intercourse with you?		
4) Actually have oral, anal, or vaginal intercourse with you?		
		
**Household dysfunction**		
*Substance abuse*	39.7	27.7
1) Live with anyone who was a problem drinker or alcoholic?		
2) Live with anyone who used street drugs?		
*Mental Illness*	26.1	20.1
1) Was a household member depressed or mentally ill?		
2) Did a household member attempt suicide?		
*Mother treated violently*	17.7	12.7
(Was your mother (or stepmother) ...)		
1) Sometimes, often, or very often pushed, grabbed, slapped, or had something thrown at her?		
2) Sometimes, often, or very often kicked, bitten, hit with a fist, or hit with something hard?		
3) Ever repeatedly hit over at least a few minutes?		
4) Ever threatened with or hurt by a knife or gun?		
*Incarcerated household member*	8.2	4.9
1) Did a household member go to prison?		
*Parental separation or divorce*	34.3	24.0
1) Were your parents ever separated or divorced?		
*Categories of adverse childhood experiences, No.*		
0	24.7	36.3
1	22.9	26.2
2	17.6	15.9
3	12.8	9.3
4 or 5	15.7	9.6
6, 7, or 8	6.3	2.7

Respondents were defined as exposed to a category if they responded "yes" to one or more of the questions in that category.

All questions used to define ACEs pertained to the respondents' first 18 years of life (≤ 18 years of age) (Table 2). Questions adapted from the Conflict Tactics Scale (CTS) [24] had 5 response categories: "never", "once or twice", "sometimes", "often", or "very often". Three types of childhood abuse were defined by Wyatt: emotional abuse (2 questions), physical abuse (2 questions), or contact sexual abuse (4 questions) [25]. We also defined 5 exposures to household dysfunction during childhood: exposure to substance abuse (defined by 2 questions)[26], mental illness (2 questions), violent treatment of mother or stepmother (4 questions)[24], criminal behaviour in the household (1 question), and parental separation or divorce (1 question). Respondents were defined as exposed to a category if they responded "yes" to 1 or more of the questions in that category.

To assess the cumulative effect of adverse childhood experiences on the risk of lung cancer, the total number of these categories of childhood exposures was summed to create the ACE score (range: 0-8) (Table 2). The statistical characteristics and validity of the ACE score and test-retest reliability of the questions have been published elsewhere [27,28]. Analyses were completed using a 6-level categorical ACE score variable (0, 1, 2, 3, 4 or 5, 6 or more ACEs) with 0 ACEs serving as the referent category.

#### Smoking behaviour

Using the complete ACE Study baseline cohort, we updated analyses by Anda and colleagues [2] that examined relationships between the number of categories of ACEs and five smoking behaviours. *Early smoking initiation *was defined as regularly smoking cigarettes by 14 years of age; *adult smoking initiation *was defined as smoking initiation at age 19 years or older; *ever smokers *were persons who had smoked at least 100 cigarettes in their lifetime; *current smokers *were those who reported smoking at the time of the survey; *heavy smokers *currently smoked 20 or more cigarettes per day. Study participants who reported that either parent smoked during the respondent's childhood were considered to have a parental history of smoking.

### Lung cancer case ascertainment during follow-up

Two methods of case ascertainment were used to identify lung cancer: *1*) incident hospitalization during follow-up that listed lung cancer on the discharge record, and 2) mortality records obtained from a search of the National Death Index that listed lung cancer as the underlying cause of death during follow-up through December 2005.

#### Incident hospitalization with lung cancer during follow-up

Up-to-date information on inpatient hospitalizations was available from Kaiser Permanente in an electronic format through 31 December 2005. Hospitalization records included a study identification number, information on the admission and discharge dates, a maximum of nine diagnosis and five procedure codes (*International Classification of Diseases, 9th Revision [ICD-9]*). Hospitalization discharge records were searched for diagnoses of lung cancer (ICD-9 code 162; *N.B*. In contrast to mortality data, hospitalization record diagnostic codes were based on ICD-9 throughout the follow-up period.) (n = 87). Study participants with a diagnosis of lung cancer located anywhere on the discharge record were considered to have been hospitalized with lung cancer. We removed records where the hospitalization occurred outside a period of valid health plan enrollment (n = 7) leaving a total of 80 hospitalizations with lung cancer among 64 study participants.

The eligible sample population from which hospitalizations were identified (n = 15,365) differed slightly from the baseline study population. A total of 724 observations were excluded from the hospitalization follow-up cohort because the baseline appointment date occurred outside of a period of enrollment in the health plan or within 120 days of a period of enrollment. The 120-day rule was incorporated to account for possible coverage by the health insurance plan under coverage continuation provided by the Consolidated Omnibus Budget Reconciliation Act of 1985 (COBRA). A total of 1248 persons were excluded from the hospitalization follow-up cohort because the ratio of time enrolled in the health plan was <80% of the total possible follow-up time. The latter exclusion was used to account for persons who were in-and-out of the health plan and therefore likely getting care through other sources that we could not identify.

Persons excluded from the hospitalization follow-up cohort were younger (18-34 yrs: 19%; 35-49: 26%; 50-64: 29%; 65-74: 19%; ≥ 75: 9%) and more likely to be nonwhite (33%), unmarried (37%), have financial problems (17%) than those who comprised the follow-up cohort (age: 18-34 yrs, 9%; 35-49, 26%; 50-64, 33%; 65-74, 22%; ≥ 75, 11%; nonwhite, 24%; unmarried, 30%; financial problems, 11%). No meaningful differences were observed by sex (men: excluded, 46%; included, 46%) or education (high school or less: excluded, 26%; included, 25%). Excluded persons were slightly more often to be current smokers (11% *v *8%) than included participants; however, there were no meaningful differences in the prevalence of a history of lung cancer, chronic obstructive pulmonary disease, asthma or tuberculosis. Finally, no meaningful difference in the distribution of ACE scores was observed between exclusion and inclusion groups, respectively (0: 36% *v *33%; 1: 26% *v *25%; 2: 16% *v *16%; 3: 9% *v *11%; 4 or 5: 10% *v *11%; 6,7, or 8: 3% *v *4%).

#### Death from lung cancer during follow-up

**Figure 1:**
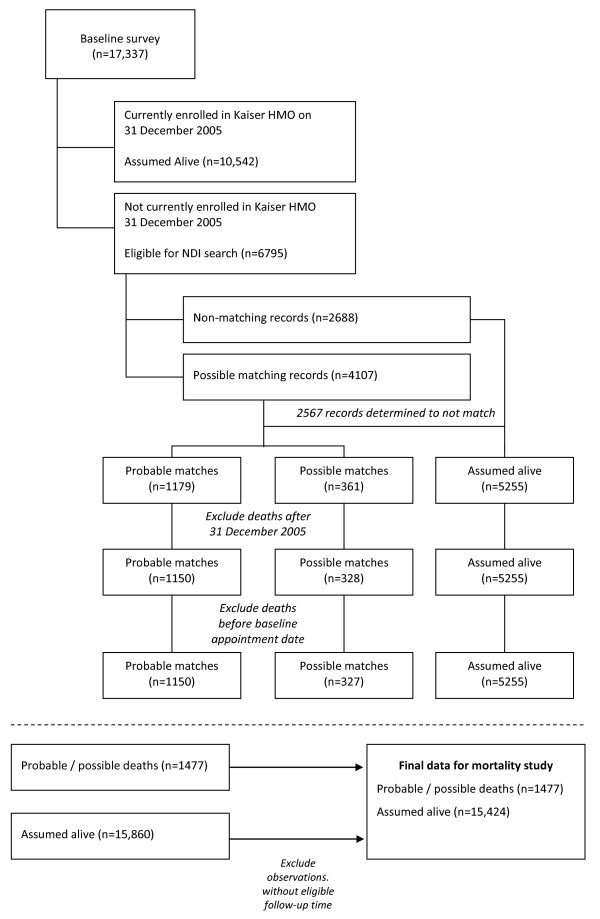
**Data map for mortality follow-up through 31 December 2005**.

To ascertain the vital status of each cohort member through 31 December 2005 (Figure 1), ACE Study baseline survey data were merged with follow-up mortality data from the National Death Index (NDI), which has been shown to capture 93-98% of all U.S. deaths [29-31]. Linkage of ACE Study participants with NDI records followed standardized procedures used by the National Center for Health Statistics [32-34]. Briefly, ACE Study participants were matched to the NDI by Social Security number, first and last names, middle initial, sex, birth date (day, month, and year), and state of residence. Eligible ACE Study participants with a "true" NDI record match were assumed to be dead, and those with no NDI record match or an NDI record match considered to be "false" match were assumed to be alive [32-34].

Of the 17,337 study participants at baseline, 10,542 were currently enrolled in the health plan on 31 December 2005 and assumed to be alive. The vital status of the remaining 6795 participants was unknown and therefore these participants were eligible for matching to the NDI. Of these 6795 participants, 4107 were identified as *potential *matching records in the NDI (Figure 1). A total of 1179 participants were identified as *probable *deaths based on an exact match between all identifying data items sent forward on the ACE Study record and on the NDI record; 361 participants were identified as *possible *deaths based in part on a probabilistic score for the match computed by NCHS and described in detail elsewhere [33]. To have comparable case ascertainment to that in the hospital discharge data, we excluded deaths after 31 December 2005 (n = 29 probable deaths; n = 33 possible matches). We identified one *possible *death record wherein the death date preceded the baseline study date and subsequently recoded this record from *possible death *to *assumed alive *leaving a total of 1477 study participants who died during follow-up (1150 probable; 327 possible).

We identified death records with an underlying cause of death of lung cancer (ICD-9 code 162 for deaths between 1995-1998 and *International Classification Disease, Tenth Revision *[ICD-10] code C34 for deaths between 1999 and 2005). The comparability ratio for ICD-9 and ICD-10 codes for lung cancer published by the National Center for Health Statistics is very high (0.9840) making analysis possible without the need to adjust for coding changes.

Follow-up (i.e., survival) time was calculated as the difference between the ACE Study baseline interview date and the last known date alive for ACE Study participants listed as decedents in the NDI and as the difference between the interview date and 31 December 2005 for those not listed as decedents. A total of 436 observations were excluded from the follow-up cohort because the baseline appointment date occurred outside of a period of enrollment in the health plan or within 120 days of a period of enrollment. Differences between study participants included and excluded from the mortality follow-up cohort were similar to those described above for hospitalization.

### Statistical analysis

Analyses were conducted using SAS v9.1.3 (2002-2003, SAS Institute, Cary, North Carolina). Associations between the number of ACE categories and each of the five smoking behaviours were examined using multivariable-adjusted logistic regression. Using multivariable-adjusted logistic regression, we estimated, by means of the odds ratio (OR), the relative risk of lung cancer occurrence during follow-up identified through hospitalization discharge records for each of the ACE score categories (1; 2; 3; 4 or 5; and 6, 7, or 8) compared to those without ACEs. Using Cox proportional hazards regression, we estimated, by means of the hazard rate ratio (HR), the relative risk of lung cancer occurrence during follow-up identified through death records across the number of categories of ACEs. We assessed the appropriateness of the proportional hazard assumption for the variables in our final model; without exception, all covariates in the final model satisfied the proportional hazard assumption.

Multivariable-adjusted models included age at baseline; sex; race/ethnicity (white, nonwhite); education (less than high school, high school graduate, some college, college graduate); marital status (married, unmarried), and current financial problems (yes, no). To assess relationships between ACEs and the occurrence of lung cancer after the addition of smoking (a causal intermediate), we included dichotomous variables for former smoking, current smoking of less than 20 cigarettes per day, and current smoking of 20 or more cigarettes per day (with never smokers as referent) as well as a measure of second hand smoke exposure (parental smoking during childhood). We also controlled for co-factors associated with an increased risk of lung cancer including a baseline history of asthma, chronic obstructive pulmonary disease (COPD), cancer, or tuberculosis.

Analysis focused on estimation of the risk of lung cancer rather than thinking in dichotomous terms of what is and is not statistically significant [35] as is done in predictive modeling.

## Results

### ACEs and Smoking Behaviour

Respondents who ever smoked were more likely to have reported experiencing the component ACEs than those who had never smoked (Table 2). However, the overall prevalence of experiencing ACEs was high at least ≥ 1 ACE reported by 75.3% of participants who had ever smoked and by 63.7% of those who had never smoked. Consistent with the findings of Anda and colleagues from Wave I ACE Study data, we observed strong, graded relationships between the number of categories of ACEs and each of the five smoking behaviours (Table 3).

**Table 3:** Association between number of categories of adverse childhood experiences (ACEs) and the prevalence and risk of selected smoking behaviors among 17,337 adults

		Early Smoking Initiation		Initiated Smoking After Age 18 yrs‡		Ever Smoked		Current Smoker		Heavy Smoker§	
		
Categories of ACEs, No.	N	Prevalence	OR(95% CI)*	Prevalence	OR (95% CI)*	Prevalence†	OR (95% CI)*	Prevalence†	OR (95% CI)*	Prevalence†	OR (95% CI)*
0	6255	3.2	1.00 (referent)	27.1	1.00 (referent)	33.7	1.00 (referent)	7.0	1.00 (referent)	2.0	1.00 (referent)
1	4514	4.9	1.53 (1.26, 1.87)	28.2	1.15 (1.04, 1.28)	39.9	1.29 (1.19, 1.40)	8.8	1.10 (0.95, 1.28)	2.5	1.12 (0.88, 1.43)
2	2758	6.1	1.88 (1.51, 2.32)	29.0	1.32 (1.17, 1.50)	45.6	1.62 (1.47, 1.78)	10.5	1.28 (1.09, 1.52)	3.6	1.38 (1.06, 1.80)
3	1650	8.2	2.69 (2.14, 3.39)	31.0	1.56 (1.34, 1.81)	50.7	1.91 (1.70, 2.14)	13.8	1.60 (1.33, 1.93)	5.0	2.05 (1.55, 2.72)
4 or 5	1690	10.3	3.55 (2.85, 4.42)	28.8	1.56 (1.33, 1.83)	55.1	2.44 (2.17, 2.74)	13.9	1.78 (1.49, 2.13)	5.4	2.39 (1.83, 3.13)
6, 7, or 8	470	17.9	7.06 (5.27, 9.45)	30.7	1.93 (1.45, 2.58)	61.4	3.27 (2.67, 4.01)	17.0	2.08 (1.59, 2.72)	6.3	2.46 (1.63, 3.71)

ACEs, adverse childhood experiences OR, odds ratio CI, confidence interval

* Logistic regression analyses include age, sex, race/ethnicity, education; *P *value for the linear trend across ACE Score categories was < 0.001 for all smoking behaviors.

† Age standardized using the 2000 Census population for California.

‡ Persons who began smoking by age 18 years were excluded from this analysis (n = 5166)

§Defined as smoking at least 1 pack of cigarettes per day (20 cigarettes per pack); light smokers were excluded from this analysis (n = 864).

### ACEs and Occurrence of Lung Cancer

#### Incident hospitalization with lung cancer during follow-up

We identified 64 cases of lung cancer during follow-up using hospital discharge records among 15,365 eligible study participants (age-standardized risk = 201 × 100,000^-1 ^population). Cases were older than those not hospitalized with lung cancer (<50 yrs: 2% *v *35%; 50-65: 42% *v *33%; ≥ 65: 56% *v *33%); more likely to be men (53% *v *46%), less likely to be nonwhite (14% *v *24%), have similar education levels (≤ high school: 27% *v *25%), more likely to be unmarried (41% *v *30%) and have similar current financial problems (11% *v *11%) were distributed similarly between persons hospitalized with lung cancer and those who were not.

The relationship of the ACE score to incident hospitalization for lung cancer was strong and graded (*P *= 0.0004) (Table 4). Compared to persons with an ACE score of 0, those with a score of 6 or more had a 3-fold increase in the risk of lung cancer (Model A: RR = 3.18, 95%CI = 0.71-14.15) (Table 4). After consideration of the causal pathway by adding smoking to the model, risk ratios were attenuated toward the null, although not completely.

**Table 4:** Frequency, age-adjusted risk, and risk ratio of the occurrence of lung cancer, identified by hospital discharge records, between baseline and 31 December 2005 by number of categories of adverse childhood experiences (ACEs) and smoking status among 15,365 adults

			Relative risk of lung cancer*	
			
	N	Hospitalized with lung cancer | Risk**	Model A RR (95% CI)	Model B RR (95% CI)
Categories of ACEs, No.				
0	5595	20 | 152.1	1.00 (referent)	1.00 (referent)
1	4030	10 | 103.8	0.73 (0.34, 1.58)	0.67 (0.31-1.45)
2	2447	11 | 195.6	1.48 (0.70, 3.10)	1.29 (0.61-2.74)
3	1428	12 | 574.4	3.10 (1.49, 6.46)	2.46 (1.17-5.19)
4 or 5	1469	9 | 433.7	2.55 (1.13, 5.74)	2.06 (0.90-4.72)
6, 7, or 8	396	2 | 347.8	3.18 (0.71, 14.15)	2.14 (0.46-9.89)
				
			*P *for trend <0.001	*P *for trend = 0.007
Smoking status				
Never	7808	7 | 58.8		1.00 (referent)
Former	6281	37 | 225.4		4.44 (1.95-10.12)
Current, <20 cig/d	772	6 | 591.4		10.27 (3.39-31.13)
Current, ≥ 20 cig/d	504	14 | 1662.8		26.97 (10.39-69.98)
				
Total	15,365	64 | 201.3		

ACEs, adverse childhood experiences RR, risk ratio CI, confidence interval

* Hospital discharge diagnosis of lung cancer defined by ICD-9 code 162

** Risk (per 100,000 population) age-standardized to the 2000 Census population for California

Model A adjusted for age, sex, race/ethnicity, education, marital status, financial problems

Model B adjusted for age, sex, race/ethnicity, education, marital status, financial problems, smoking status, parental smoking history. In addition to the RR estimates for ACE score, we show the RR estimates for smoking status from the regression model.

As the ACE score increased, the adjusted mean age at incident hospitalization for lung cancer decreased (*P *for trend < 0.001). Persons with 6 or more ACEs were hospitalized 13 years earlier on average than those without ACEs (60.7 years; 95%CI = 49.2-72.3 *v *73.8 years; 95%CI = 70.3-77.4). Of course, comparisons of average-at-hospitalization across groups are not straightforward since the average age-at-hospitalization depends to a large extent on the age distribution of the underlying groups being compared.

#### Death from lung cancer during follow-up

The 16,901 study participants eligible for mortality follow-up contributed 120,562 years of person-time (average = 7.1 years). Using death records, we identified 111 cases of lung cancer (age-standardized mortality rate = 31.1 × 100,000^-1 ^person-years). (*N.B*. Age-stratified risk of lung cancer mortality by ACE score is shown in Additional file 1.) Cases were older (<50 yrs: 2% *v *36%; 50-65: 30% *v *32%; ≥ 65: 68% *v *32%) and more often men (57% *v *46%), white (87% *v *75%), and less educated (≤ high school: 41% *v *25%) than those who survived follow-up (or were censored); the proportion of unmarried persons (28% *v *31%) and those with financial problems (11% *v *12%) were similar across groups.

Risk ratios, estimated by the hazard rate ratio, for the occurrence of lung cancer were modestly increased across the number of categories of ACEs with the exception of that for persons with 6 or more ACEs for whom the risk ratio was 3.55 (95%CI = 1.25-10.09) (Table 5). Risk ratios were attenuated toward the null after addition of smoking to the model. A possible association remained between ACE scores of 6 or more and lung cancer although a small number of cases (n = 4) among the exposed pose a challenge to interpretation.

**Table 5:** Frequency, age-adjusted risk, and risk ratio of the occurrence of lung cancer, identified by death records, between baseline and 31 December 2005 by number of categories of adverse childhood experiences (ACEs) and smoking status among 16,901 adults

				Relative risk of lung cancer*	
				
	N	Cases	Age-adjusted risk (95% CI)**	Model A RR (95% CI)	Model B RR (95% CI)
Categories of ACEs, No.					
0	6124	53	359.4 (268.7-480.6)	1.00 (referent)	1.00 (referent)
1	4411	26	248.8 (168.9-366.3)	0.75 (0.47, 1.20)	0.69 (0.43, 1.11)
2	2681	28	720.5 (394.8-1311.0)	1.52 (0.95, 2.42)	1.35 (0.84, 2.16)
3	1599	18	805.5 (492.8-1313.9)	1.92 (1.11, 3.33)	1.58 (0.90, 2.76)
4 or 5	1637	15	641.0 (373.9-1096.6)	1.88 (1.04, 3.41)	1.51 (0.83, 2.78)
6, 7, or 8	449	4	635.8 (239.5-1676.8)	2.70 (0.94, 7.72)	1.83 (0.63, 5.35)
					
				*P *for trend = 0.001	*P *for trend = 0.017
Smoking status					
Never	8589	16	108.4 (64.9-179.8)		1.00 (referent)
Former	6879	90	539.6 (426.0-683.2)		4.83 (2.80-8.33)
Current, <20 cig/d	870	13	1166.8 (676.2-2006.2)		10.11 (4.78-21.39)
Current, ≥ 20 cig/d	563	25	3448.5 (2210.2-5342.7)		25.48 (13.10-49.56)
					
Total	16,901	144	432.3 (362.2-515.7)		

ACEs, adverse childhood experiences RR, risk ratio CI, confidence interval

* Lung cancer cases identified through either a hospital discharge diagnosis of lung cancer defined by ICD-9 code 162 or an underlying cause of death from lung cancer defined by ICD-9 code 162 for deaths between 1995-1998; ICD-10 code C34 for deaths between 1999 and 2005.

** Rate (per 100,000 population) age-standardized to the 2000 Census population for California.

Model A adjusted for age, sex, race/ethnicity, education, married, financial problems

Model B adjusted for age, sex, race/ethnicity, education, married, financial problems, smoking status, parental smoking history. In addition to the RR estimates for ACE score, we show the RR estimates for smoking status from the regression model.

We combined cases from the two prospective case ascertainment methods and observed 144 cases of lung cancer (age-standardized risk = 432.3 × 100,000^-1 ^population) (Table 6). The relationship of the ACE score to the risk of lung cancer was strong and graded (*P *= 0.001). Similar patterns were observed to those described above. The age-adjusted risk difference, comparing persons with ACE scores of 6 or more to those without ACEs, was 277 cases × 100,000^-1 ^population and risk ratios were about 1.5-2.5 times greater for persons with 3 or more categories of ACEs compared to those without ACEs. As observed above, risk ratios were attenuated toward the null, although not completely, after addition of smoking to the model. Similar findings were observed after further addition of a baseline history of asthma, COPD, cancer, or tuberculosis (ACE score = 1: RR = 0.70, 95%CI = 0.45-1.14; ACE score = 2: RR = 1.34, 95%CI = 0.83-2.15; ACE score = 3: RR = 1.57, 95%CI = 0.90-2.76; ACE score = 4 or 5: RR = 1.40, 95%CI = 0.76-2.58; ACE score = 6, 7, or 8: RR = 1.70, 95%CI = 0.58-4.97).

**Table 6:** Frequency, age-adjusted risk, and risk ratio of the occurrence of lung cancer, identified by hospital or death records, between baseline and 31 December 2005 by number of categories of adverse childhood experiences (ACEs) and smoking status among 16,901 adults

				Relative risk of lung cancer*	
				
	N	Cases	Age-adjusted risk (95% CI)**	Model A RR (95% CI)	Model B RR (95% CI)
Categories of ACEs, No.					
0	6124	53	359.4 (268.7-480.6)	1.00 (referent)	1.00 (referent)
1	4411	26	248.8 (168.9-366.3)	0.75 (0.47, 1.20)	0.69 (0.43, 1.11)
2	2681	28	720.5 (394.8-1311.0)	1.52 (0.95, 2.42)	1.35 (0.84, 2.16)
3	1599	18	805.5 (492.8-1313.9)	1.92 (1.11, 3.33)	1.58 (0.90, 2.76)
4 or 5	1637	15	641.0 (373.9-1096.6)	1.88 (1.04, 3.41)	1.51 (0.83, 2.78)
6, 7, or 8	449	4	635.8 (239.5-1676.8)	2.70 (0.94, 7.72)	1.83 (0.63, 5.35)
					
				*P *for trend = 0.001	*P *for trend = 0.017
Smoking status					
Never	8589	16	108.4 (64.9-179.8)		1.00 (referent)
Former	6879	90	539.6 (426.0-683.2)		4.83 (2.80-8.33)
Current, <20 cig/d	870	13	1166.8 (676.2-2006.2)		10.11 (4.78-21.39)
Current, ≥ 20 cig/d	563	25	3448.5 (2210.2-5342.7)		25.48 (13.10-49.56)
					
Total	16,901	144	432.3 (362.2-515.7)		

ACEs, adverse childhood experiences RR, risk ratio CI, confidence interval

* Lung cancer cases identified through either a hospital discharge diagnosis of lung cancer defined by ICD-9 code 162 or an underlying cause of death from lung cancer defined by ICD-9 code 162 for deaths between 1995-1998; ICD-10 code C34 for deaths between 1999 and 2005.

** Rate (per 100,000 population) age-standardized to the 2000 Census population for California.

Model A adjusted for age, sex, race/ethnicity, education, married, financial problems

Model B adjusted for age, sex, race/ethnicity, education, married, financial problems, smoking status, parental smoking history. In addition to the RR estimates for ACE score, we show the RR estimates for smoking status from the regression model.

#### Premature death from lung cancer

Following on prior analyses suggesting associations between ACEs and premature all-cause mortality [36], we repeated analyses for premature death from lung cancer. Among those who died from lung cancer, persons with 6 or more ACEs died nearly 13 years earlier on average (62.0 years; 95%CI = 53.7-70.2) on average than those without ACEs (75.4 years; 95%CI = 73.0-77.8). We re-ran the models in Table 5 after redefining the outcome as time to death from lung cancer at age 65 years or before (n = 10 deaths) and age 75 years or before (n = 55 deaths). Comparing persons with 6 or more ACEs to those without ACEs, risk ratios for the occurrence of lung cancer were 10.48 (95%CI = 1.94-56.64) (Model A, adjusted for age, sex, race/ethnicity, education, marital status, and financial problems) and 7.90 (95%CI = 1.40-44.61) (Model B, adjusted for age, sex, race/ethnicity, education, marital status, financial problems, smoking status, parental smoking history) for death at age 65 or before; 4.72 (95%CI = 1.54-14.44) (Model A) and 2.90 (95%CI = 0.92-9.11) (Model B) for death at age 75 or before.

## Discussion and Conclusion

Using prospective data we observed graded relationships between the ACE score and the risk of lung cancer. Moreover, relationships between a high ACE score and lung cancer were particularly strong for those who died from lung cancer at younger ages. The increase in risk of lung cancer was only partly due to relationships between ACEs and an intermediate causal factor, smoking. The occurrence of ACE-related lung cancer not attributable to conventional risk factors suggests other mechanisms by which childhood traumatic stressors negatively affect health.

The observed associations between ACEs and lung cancer may be conservative. Case fatality for lung cancer is high. The overall 5-year relative survival rate for 1996-2004 from 17 Surveillance Epidemiology and End Results (SEER) geographic areas in the United States was 15% (age <65 years, 18%; age >/=65 years, 13%) with a survival rate for small cell lung cancer of about 6% and for non-small cell of only 17% [37]. Thus, given relationships between ACEs and smoking behaviours (particularly associations with early smoking initiation) which would increase the probability of developing smoking-related disease, it is possible that some Kaiser members with higher ACE scores were less likely to survive and to be included in the baseline data collection because they had already died from lung cancer or another smoking-related disease.

Some degree of selection bias is inevitable in observational research simply because not all persons who are born will survive to the observation period of interest and because the population that does survive often differs from the population that does not. In the case of ACEs, which are associated with numerous adverse health behaviours and health outcomes (perhaps most importantly premature death), it is reasonable to postulate that persons who are exposed to ACEs (particularly multiple ACEs) are more likely than those who are not exposed to die during childhood or young adulthood, be institutionalized, or otherwise lost prior to the initiation of the ACE Study and baseline survey resulting in a downward bias for the association between ACEs and lung cancer. Some caution must be exercised in making such an assertion with regard to the direction of the bias since this does not always hold for non-dichotomous exposures.

A strength of this study lies in the use of two prospective data sources to identify cases of lung cancer. The prospective data from hospital and mortality records are not subject to recall bias and are reported by physicians who were unaware of the patient ACE score. Also, the ACE Study incorporates multiple forms of childhood traumatic stressors. Studies that examine only one or at most two types of stressors may *1*) underestimate the burden of exposure, *2*) fail to recognize the interrelationships between different types of traumatic stressors during childhood, and/or *3*) incorrectly attribute long-term consequences to single types of childhood traumatic stress [38] despite convincing evidence suggesting that exposure to multiple forms of abuse and traumatic stressors appear to influence health behaviors and outcomes through a cumulative process.

The results of this study are subject to several limitations. The frequency of ACEs may represent an underreporting of their actual occurrence given the sensitive nature of the questions. However, our estimates of the prevalence of childhood exposures are similar to estimates from nationally representative surveys [39,40] indicating that the experiences of our participants are comparable to those of the larger population of adults. For example, in our study we found that 16% of the men and 25% of the women met the case definition for contact sexual abuse; a national telephone survey of adults in US conducted by Finkelhor and colleagues [41] using similar criteria for sexual abuse estimated that 16% of men and 27% of women had been sexually abused. Of the men from our study, 30% had been physically abused as boys, which closely parallels the percentage (31%) found in a recent population-based study of Ontario men in Canada that used questions from the same scales [42]. The similarity in estimates of the prevalence of these childhood exposures between the ACE Study and other population-based studies suggests that our findings are likely to be applicable in other settings.

The adverse effects of smoking are in part a function of the amount smoked, duration of smoking, inhalation, and tobacco product smoked. While we were able to incorporate the amount smoked, this analysis did not have data on duration and therefore was not able to compute the number of pack-years smoked. Thus, associations between ACEs and the occurrence of lung cancer that remained after the addition of smoking into the model may be the result of our inability to capture pathway effects of smoking duration. Also, smoking status was based on a single measure at baseline; therefore, we do not have data on initiation or cessation during follow-up. Similarly, exposure to second hand smoke may have changed over time. While we included variables in the final model for baseline prevalent asthma, COPD, and tuberculosis - conditions associated with the occurrence of lung cancer - we did not have information on occupational or other environmental exposures (e.g., asbestos, radon).

ACEs are associated with risk factors for chronic disease conditions such as ischemic heart disease [43], liver disease [44], COPD [45] and mental disorders [46,47] that may result in an increased risk of exacerbating underlying lung disease and/or negatively affect general health, leading to disease progression or perhaps increasing the likelihood of undiagnosed lung cancer being identified [45]. Although mortality follow-up was available for a maximum of 10 years, statistical power was somewhat limited owing to relatively few deaths during follow-up among persons exposed to multiple ACEs. We plan to continue repeating the NDI search and related analyses in the coming years. As is the case in many observational studies, there may have been unknown or unmeasured confounding factors for which adjustment was not possible. Moreover, measurement error in the assessment or estimation of covariates and their severity may have resulted in incomplete adjustment and residual confounding. We feel these data are compatible with a moderate association between ACEs and risk of lung cancer; however, this assumes that there is no bias in the data collected and that our statistical models are correct [48].

Finally, we examined competing risks as a potential explanation for observed results using mortality data. If competing causes of loss to follow-up act independent of the outcome (e.g., lung cancer), then consistent estimates of the survival function are possible. Alternatively, if the independence assumption does not hold, a bias can be introduced because the number of failures from the competing risk may influence the number of subjects at risk for the outcome of interest. After identifying deaths during follow-up from smoking-related diseases [49] (other than lung cancer) (n = 707 deaths) and removing these observations from the censored group, we repeated the models shown in Table 5 and observed similar results for risk of lung cancer death at any age as well as premature death from lung cancer.

In summary, exposure to adverse childhood experiences is common. Insofar as stressful and traumatic childhood experiences contribute to the adoption of adverse health behaviours, such as smoking, and subsequent development of poor health outcomes, such as death from lung cancer, these childhood exposures should be recognized as underlying causes of premature mortality [50]. Reducing the burden of adverse childhood experiences should be considered in health and social programs as a means of primary prevention of lung cancer as well as other smoking-related diseases [43,45]. In addition, because smoking did not completely explain observed relationships between ACEs and the occurrence of lung cancer, other pathophysiologic pathways by which childhood stressors may influence the risk of lung cancer should be explored.

## Competing interests

The authors declare that they have no competing interests.

## Authors' contributions

Study conception and design: DWB, RFA. Acquisition of data: RFA, VJF, VJE. Analysis and interpretation of data: DWB, RFA, JBC. Drafting of manuscript: DWB, RFA, VJF, VJE, AMM, JBC, WHG. Critical revision: DWB, RFA, JBC. All authors read and approved the final manuscript.

## Pre-publication history

The pre-publication history for this paper can be accessed here:

http://www.biomedcentral.com/1471-2458/10/20/prepub

## Supplementary Material

Additional file 1 **Table A1**. Risk of death from lung cancer (× 1000_-1 _population) by age and number of categories of adverse childhood experiences. Click here for file

## References

[B1] Felitti VJ Anda RF Nordenberg D Relationship of childhood abuse and household dysfunction to many of the leading causes of death in adults. The Adverse Childhood Experiences (ACE) Study Am J Prev Med 1998 14 245 58 10.1016/S0749-3797(98)00017-8 9635069

[B2] Anda RF Croft JB Felitti VJ Adverse childhood experiences and smoking during adolescence and adulthood JAMA 1999 282 1652 8 10.1001/jama.282.17.1652 10553792

[B3] Jun HJ Rich-Edwards JW Boynton-Jarrett R Child abuse and smoking among young women: the importance of severity, accumulation, and timing J Adolesc Health 2008 43 55 63 10.1016/j.jadohealth.2007.12.003 18565438 PMC3932335

[B4] Carmody TP Affect regulation, nicotine addiction, and smoking cessation J Psychoactive Drugs 1992 24 111 22 1506996 10.1080/02791072.1992.10471632

[B5] Gehricke JG Loughlin SE Whalen CK Smoking to self-medicate attentional and emotional dysfunctions Nicotine Tob Res 2007 9 Suppl 4 S523 36 10.1080/14622200701685039 18067030

[B6] Benowitz NL Clinical pharmacology of nicotine: implications for understanding, preventing, and treating tobacco addiction Clin Pharmacol Ther 2008 83 531 41 10.1038/clpt.2008.3 18305452

[B7] Anda RF Felitti VJ Bremner JD The enduring effects of abuse and related adverse experiences in childhood: a convergence of evidence from neurobiology and epidemiology Eur Arch Psychiatry Clin Neurosci 2006 256 174 86 10.1007/s00406-005-0624-4 16311898 PMC3232061

[B8] Fuller-Thomson E Brennenstuhl S Making a link between childhood physical abuse and cancer: results from a regional representative survey Cancer 2009 115 3341 50 10.1002/cncr.24372 19472404

[B9] Hertzman C The biological embedding of early experience and its effects on health in adulthood Ann N Y Acad Sci 1999 896 85 95 10.1111/j.1749-6632.1999.tb08107.x 10681890

[B10] Teicher MH Andersen SL Polcari A The neurobiological consequences of early stress and childhood maltreatment Neurosci Biobehav Rev 2003 27 33 44 10.1016/S0149-7634(03)00007-1 12732221

[B11] Bremmer JD Long-term effects of childhood abuse on brain and neurobiology Child Adolesc Psychiatric Clin N Am 2003 12 271 92 10.1016/S1056-4993(02)00098-6 12725012

[B12] Bremmer JD Alterations in brain structure and function associated with post-traumatic stress disorder Semin Clin Neuropsychiatry 1999 4 249 55 10553030 10.153/SCNP00400249

[B13] Heim C Newport DJ Wagner D The role of early adverse experience and adulthood stress in the prediction of neuroendocrine stress reactivity in women: a multiple regression analysis Depress Anxiety 2002 15 117 25 10.1002/da.10015 12001180

[B14] Coussens LM Werb Z Inflammation and cancer Nature 2002 420 860 7 10.1038/nature01322 12490959 PMC2803035

[B15] Walser T Cui X Yanagawa J Smoking and lung cancer: the role of inflammation Proc Am Thorac Soc 2008 5 811 5 10.1513/pats.200809-100TH 19017734 PMC4080902

[B16] Seruga B Zhang H Bernstein LJ Tannock IF Cytokines and their relationship to the symptoms and outcome of cancer Nat Rev Cancer 2008 8 887 99 10.1038/nrc2507 18846100

[B17] Bhalla DK Hirata F Rishi AK Gairola CG Cigarette smoke, inflammation, and lung injury: a mechanistic perspective J Toxicol Environ Health B Crit Rev 2009 12 45 64 19117209 10.1080/10937400802545094

[B18] U.S. Department of Health and Human Services The Health Consequences of Smoking: A Report of the Surgeon General 2004 Atlanta, GA: U.S. Department of Health and Human Services, Centers for Disease Control and Prevention, National Center for Chronic Disease Prevention and Health Promotion, Office on Smoking and Health

[B19] U.S. Department of Health and Human Services The Health Consequences of Involuntary Exposure to Tobacco Smoke: a Report of the Surgeon General 2006 Atlanta, GA: U.S. Dept. of Health and Human Services, Centers for Disease Control and Prevention, Coordinating Center for Health Promotion, National Center for Chronic Disease Prevention and Health Promotion, Office on Smoking and Health

[B20] Centers for Disease Control and Prevention (CDC) Smoking-attributable mortality, years of potential life lost, and productivity losses -- United States, 2000-2004 MMWR Morb Mortal Wkly Rep 2008 57 1226 8 19008791

[B21] World Health Organization WHO Report on the Global Tobacco Epidemic, 2008: the MPOWER package 2008 Geneva, Switzerland. WHO Press

[B22] Centers for Disease Control and Prevention, National Center for Health Statistics Compressed Mortality File 1999-2005 CDC WONDER On-line Database, compiled from Compressed Mortality File 1999-2005 Series 20 No. 2K 2008

[B23] Dube SR Anda RF Felitti VJ Growing up with parental alcohol abuse: exposure to childhood abuse, neglect, and household dysfunction Child Abuse Negl 2001 25 1627 40 10.1016/S0145-2134(01)00293-9 11814159

[B24] Straus M Gelles RJ Physical Violence in American Families: Risk Factors and Adaptations to Violence in 8,145 Families 1990 New Brunswick, NJ: Transaction Press

[B25] Wyatt GE The sexual abuse of Afro-American and white-American women in childhood Child Abuse Negl 1985 9 507 19 10.1016/0145-2134(85)90060-2 4084830

[B26] Schoenborn CA Exposure to alcoholism in the family: United States, 1988 Adv Data 1991 205 1 13 10114780

[B27] Dong M Anda RF Felitti VJ The interrelatedness of multiple forms of childhood abuse, neglect, and household dysfunction Child Abuse Negl 2004 28 771 84 10.1016/j.chiabu.2004.01.008 15261471

[B28] Dube SR Williamson DF Thompson T Felitti VJ Anda RF Assessing the reliability of retrospective reports of adverse childhood experiences among adult HMO members attending a primary care clinic Child Abuse Negl 2004 28 729 37 10.1016/j.chiabu.2003.08.009 15261468

[B29] Calle EE Terrell DD Utility of the National Death Index for ascertainment of mortality among Cancer Prevention Study II participants Am J Epidemiol 1993 137 235 41 8452128 10.1093/oxfordjournals.aje.a116664

[B30] Edlavitch SA Baxter J Comparability of mortality follow-up before and after the National Death Index Am J Epidemiol 1988 127 1164 78 3369416 10.1093/oxfordjournals.aje.a114910

[B31] Stampfer MJ Willett WC Speizer FE Test of the National Death Index Am J Epidemiol 1984 119 837 9 6720679 10.1093/oxfordjournals.aje.a113804

[B32] National Center for Health Statistics National Death Index User's Manual 2003 Hyattsville, Maryland: National Center for Health Statistics Available upon request from NCHS

[B33] Horm J Multiple causes of death for the National Health Interview Survey Committee on Applied and Theoretical Statistics, National Research Council, Federal Committee on Statistical Methodology, Office of Management and Budget. Record Linkage Techniques--1997 Proceedings of an International Workshop and Exposition 1999 National Academy Press: Washington, DC 71 77

[B34] National Center for Health Statistics Office of Analysis and Epidemiology The 1986-2000 National Health Interview Survey linked mortality files: matching methodology 2005 Hyattsville, Maryland: National Center for Health Statistics http://www.cdc.gov/nchs/data/datalinkage/matching_methodology_nhis_final.pdf

[B35] Rothman KJ Lessons from John Graunt Lancet 2006 347 37 9 10.1016/S0140-6736(96)91562-7 8531550

[B36] Brown DW Anda RF Tiemeier H Adverse childhood experiences and the risk of premature death Am J Prev Med 2009 37 389 96 10.1016/j.amepre.2009.06.021 19840693

[B37] National Cancer Institute Surveillance Epidemiology and End Results Stat Fact Sheet http://seer.cancer.gov/statfacts/html/lungb.html on 20 January 2009

[B38] Finkelhor D Ormrod R Turner H Hamby SL The victimization of children and youth: a comprehensive, national survey Child Maltreat 2005 10 5 25 10.1177/1077559504271287 15611323

[B39] Finkelhor D Dziuba-Leatherman J Children as victims of violence: A national survey Pediatrics 1994 94 413 20 7936846

[B40] Wyatt GE Loeb TB Solis B Carmona JV Romero G The prevalence and circumstances of child sexual abuse: Changes across a decade Child Abuse Negl 1999 23 45 60 10.1016/S0145-2134(98)00110-0 10075192

[B41] Finkelhor D Hotaling G Lewis IA Smith C Sexual abuse in a national survey of adult men and women: Prevalence, characteristics, and risk factors Child Abuse Negl 1990 14 19 28 10.1016/0145-2134(90)90077-7 2310970

[B42] MacMillan HL Fleming JE Trocme N Prevalence of child physical and sexual abuse in the community results from the Ontario health supplement JAMA 1997 278 131 5 10.1001/jama.278.2.131 9214528

[B43] Dong M Giles WH Felitti VJ Insights into causal pathways for ischemic heart disease: Adverse Childhood Experiences Study Circulation 2004 110 1761 6 10.1161/01.CIR.0000143074.54995.7F 15381652

[B44] Dong M Dube SR Felitti VJ Giles WH Anda RF Adverse childhood experiences and self-reported liver disease: new insights into the causal pathway Arch Intern Med 2003 163 1949 56 10.1001/archinte.163.16.1949 12963569

[B45] Anda RF Brown DW Dube SR Adverse childhood experiences and chronic obstructive pulmonary disease in adults Am J Prev Med 2008 34 396 403 10.1016/j.amepre.2008.02.002 18407006 PMC8214869

[B46] Chapman DP Whitfield CL Anda RF Epidemiology of adverse childhood experiences and depressive disorders in a large health maintenance organization population J Affect Disord 2004 82 217 25 10.1016/j.jad.2003.12.013 15488250

[B47] Anda RF Brown DW Felitti VJ Adverse childhood experiences and prescribed psychotropic medications in adulthood: a prospective study Am J Prev Med 2007 32 389 94 10.1016/j.amepre.2007.01.005 17478264 PMC3233770

[B48] Rothman KJ Greenland S Lash TL Modern Epidemiology 2008 3 Philadelphia, Pennsylvania: Lippincott Williams & Wilkins 151 167

[B49] Centers for Disease Control and Prevention Smoking-Attributable Mortality, Morbidity, and Economic Costs (SAMMEC): Adult SAMMEC and Maternal and Child Health (MCH) SAMMEC software, 2007 http://apps.nccd.cdc.gov/sammec/

[B50] Anda RF Brown DW Root causes and organic budgeting: funding health from conception to the grave Ped Health 2007 1 141 3 10.2217/17455111.1.2.141

