# Adverse childhood experiences are associated with the risk of lung cancer: a prospective cohort study

**DOI:** 10.1186/1471-2458-10-311

**Published:** 2010-06-04

**Authors:** David W Brown, Robert F Anda, Vincent J Felitti, Valerie J Edwards, Ann Marie Malarcher, Janet B Croft, Wayne H Giles

**Affiliations:** 1 Centers for Disease Control and Prevention, Atlanta, Georgia, USA; 2 Netherlands Institute for Health Sciences, Erasmus University Medical Center, Rotterdam, the Netherlands; 3 Southern California Permanente Group (Kaiser Permanente), San Diego, California, USA

## Correction

After the publication of this work [1], we became aware that Table 5 (Table 1)  incorrectly duplicated the information in Table 6. We have corrected this error and have inserted the correct Table 5 (Table 1) below.

**Table 5:** Frequency, age-adjusted risk and risk ratio of the occurrence of lung cancer, identified by death records, between baseline and 31 December 2005 by number of categories of adverse childhood experiences (ACEs) and smoking status among 16,901 adults

					Relative risk of lung cancer*	
	N |	Person- Time (yrs)	Deaths |	Mortality Rate**	Model A RR (95% CI)	Model B RR (95% CI)
Categories of ACEs, No.						
0	6124 |	44,592	44 |	26.0	1.00 (referent)	1.00 (referent)
1	4411 |	31,709	22 |	19.3	0.80 (0.48-1.34)	0.74 (0.44, 1.23)
2	2681 |	19,045	22 |	66.4	1.48 (0.89-2.49)	1.30 (0.77, 2.18)
3	1599 |	11,056	11 |	51.3	1.52 (0.78-2.96)	1.17 (0.59, 2.29)
4 or 5	1637 |	11,259	8 |	30.0	1.32 (0.61-2.83)	1.00 (0.46, 2.16)
6, 7, or 8	449 |	2901	4 |	62.7	3.55 (1.25-10.09)	2.34 (0.81, 6.75)
						
					*P *for trend = 0.025	*P *for trend = 0.251
						
Smoking status						
Never	8589 |	61,713	11 |	8.5		1.00 (referent)
Former	6879 |	49,379	73 |	58.0		5.92 (3.10-11.30)
Current,<20 cig/d	870 |	5748	10 |	129.7		12.43 (5.23-29.57)
Current, >20 cig/d	563 |	3722	17 |	357.1		28.15 (12.77-62.08)
Total	16,901 |	120,562	111 |	31.1		

ACEs, adverse childhood experiences RR, risk ratio CI, confidence interval

* Underlying cause of death from lung cancer defined by ICD-9 code 162 for deaths between 1995-1998; ICD-10 code C34 for deaths between 1999 and 2005.

** Rate (per 100,000 person-years) age-standardized to the 2000 Census population for California.

Model A adjusted for age, sex, race/ethnicity, education, married, financial problems

Model B adjusted for age, sex, race/ethnicity, education, married, financial problems, smoking status, parental smoking history. In addition to the RR estimates for ACE score, we show the RR estimates for smoking status from the regression model.

We regret any inconvenience that this oversight may have caused.

## Pre-publication history

The pre-publication history for this paper can be accessed here:

http://www.biomedcentral.com/1471-2458/10/311/prepub
