# Supplementary material for: Adverse childhood experiences are associated with the risk of lung cancer: a prospective cohort study
Source: BMC Public Health. 2010 Jan 19;10:20. doi: 10.1186/1471-2458-10-20 (PMC2826284; doi:10.1186/1471-2458-10-20)
Supplement: Additional file 1 — Table A1. Risk of death from lung cancer (× 1000-1 population) by age and number of categories of adverse childhood experiences. [file 1471-2458-10-20-S1.PDF]

Annex

Table A1. Risk of death from lung cancer (x 1000^{-1} population) by age and number of categories of adverse childhood experiences

Age 18-54

ACE Score	D+	D–	Risk
0	2	2262	0.9
1	0	1907	0.0
2	1	1344	0.7
3	1	893	1.1
4 or 5	2	1044	1.9
6, 7, or 8	2	334	6.0
Total	8	7784	

Age 55-64

ACE Score	D+	D–	Risk
0	8	1267	6.3
1	9	984	9.1
2	4	579	6.9
3	2	353	5.6
4 or 5	2	326	6.1
6, 7, or 8	2	77	25.3
Total	27	3586	

Age 65-74

ACE Score	D+	D–	Risk
0	21	1600	13.0
1	7	992	7.0
2	12	513	22.9
3	4	265	14.9
4 or 5	4	202	19.4
6, 7, or 8	0	29	0.0
Total	48	3601	

Age 75-84

ACE Score	D+	D–	Risk
0	13	861	14.9
1	6	475	12.5
2	4	215	18.3
3	4	70	54.1
4 or 5	0	54	0.0
6, 7, or 8	0	4	0.0
Total	27	1679	

Age 85+

ACE Score	D+	D–	Risk
0	0	90	0.0
1	0	31	0.0
2	1	8	111.1
3	0	7	0.0
4 or 5	0	3	0.0
6, 7, or 8	0	1	0.0
Total	1	140	
